# Machine Learning for Predicting Mycotoxin Occurrence in Maize

**DOI:** 10.3389/fmicb.2021.661132

**Published:** 2021-04-09

**Authors:** Marco Camardo Leggieri, Marco Mazzoni, Paola Battilani

**Affiliations:** Department of Sustainable Crop Production, Università Cattolica del Sacro Cuore, Piacenza, Italy

**Keywords:** aflatoxins, *Aspergillus flavus*, cropping system, deep learning, *Fusarium verticillioides*, fumonisins, predictive models

## Abstract

Meteorological conditions are the main driving variables for mycotoxin-producing fungi and the resulting contamination in maize grain, but the cropping system used can mitigate this weather impact considerably. Several researchers have investigated cropping operations’ role in mycotoxin contamination, but these findings were inconclusive, precluding their use in predictive modeling. In this study a machine learning (ML) approach was considered, which included weather-based mechanistic model predictions for AFLA-maize and FER-maize [predicting aflatoxin B_1_ (AFB_1_) and fumonisins (FBs), respectively], and cropping system factors as the input variables. The occurrence of AFB_1_ and FBs in maize fields was recorded, and their corresponding cropping system data collected, over the years 2005–2018 in northern Italy. Two deep neural network (DNN) models were trained to predict, at harvest, which maize fields were contaminated beyond the legal limit with AFB_1_ and FBs. Both models reached an accuracy >75% demonstrating the ML approach added value with respect to classical statistical approaches (i.e., simple or multiple linear regression models). The improved predictive performance compared with that obtained for AFLA-maize and FER-maize was clearly demonstrated. This coupled to the large data set used, comprising a 13-year time series, and the good results for the statistical scores applied, together confirmed the robustness of the models developed here.

## Introduction

Mycotoxin contamination of maize is a major concern worldwide (Eskola et al., 2020). The colonization of maize ears by *Aspergillus* section *Flavi* and *Fusarium* spp. can lead to ear rots whose impact on the amount of grain yield is minor or negligible yet their mycotoxin contamination levels are high; therefore, the mains impact of mycotoxin producing fungi in maize regards grain safety and its compliance with the legal limits. Concerning those mycotoxins produced by *Aspergillus* section *Flavi*, among the aflatoxins (AFs), aflatoxin B_1_ (AFB_1_) is classified by IARC (International Agency for Research on Cancer) as a class-1A, human carcinogen. Such AFs were first detected in Italy in the early 2000s (Piva et al., 2006; Battilani et al., 2008a), but since 2012 they have spread all over southeastern Europe, presumably aided by warmer and drier conditions during summer attributed to ongoing climate change (Dobolyi et al., 2013; Levic et al., 2013; Battilani et al., 2016), and their incidence and severity can vary markedly among years. *Fusarium* spp. can produce a wide range of mycotoxins, of which the fumonisins B_1_, B_2_, and B_3_ (FBs)—predominantly produced by *F. verticillioides*—are the key ones reported in maize grain worldwide, thus posing a serious risk of possible human carcinogenicity (IARC, 1993). Other mycotoxins produced by *Fusarium* genus are the trichothecenes (TCTs) and zearalenone (ZEN), these being prevalent in temperate and wet areas especially in rainy years, optimal conditions for their main producer, *F. graminearum* (Pietri et al., 2004).

These main mycotoxins threaten the maize supply chain worldwide, in all producing areas; nevertheless, the prevailing mycotoxin and level of contamination depends both on the growing area and year, intended as the meteorlogical conditions occurring during the crop growing season (Logrieco et al., 2021). Support for farmers coming from predictive modeling, using meteorological data as input variables (Battilani, 2016), has been pursued in Europe in the form of two mechanistic models for AFB_1_ and FBs predictions: respectively, AFLA-maize (Battilani et al., 2013) and FER-maize (Battilani et al., 2003). They both aim to predict the risk of contamination above current legal limits in force in Europe (European Commission, 2006b, 2007), and they strongly support stakeholders in the maize chain management (Battilani and Camardo Leggieri, 2015; Battilani, 2016; Palumbo et al., 2020). However, mounting uncertainty of climate conditions and extreme events, often emphasized as issues in climate change, has recently increased the importance of deriving reliable predictions at the farm level (Camardo Leggieri et al., 2020a). Addressing the variability in mycotoxin occurrence among years and geographic areas, even those quite close to each other, in addition to the emerging issue of co-occurring mycotoxins (Camardo Leggieri et al., 2019; Giorni et al., 2019), will require making reliable predictions to support the maize chain management.

Weather variables are the leading factors contributing to mycotoxin occurrence, but the cropping system used is a powerful tool of farmers to mitigate grain contamination. Accordingly, several authors have studied the role of the cropping system and the rationale behind its impact on mycotoxin contamination (Munkvold, 2003; Battilani et al., 2008a; Blandino et al., 2009; Kos et al., 2013; Palumbo et al., 2020). A rationale crop rotation, leaving the land fallow (unsown with maize), is recommended to reduce mycotoxin contamination in maize fields, even if the impact on it cannot be readily demonstrated, especially in intensive maize-growing areas (Guo et al., 2005; Marocco et al., 2008; Munkvold, 2014). A significant impact of the season length of maize hybrids, frequently reported as FAO class (Food and Agriculture Organization classification), upon FBs and AFB_1_ contamination was reported (Pietri and Bertuzzi, 2012). Scientist do not all agree on this statement (Battilani et al., 2008a; Mazzoni et al., 2011), but the number of days elapsed from sowing to harvest was positively related to mycotoxin contamination (Battilani et al., 2008a; Torelli et al., 2012). The sowing date has been confirmed to influence the likelihood and extent of mycotoxin contamination (Jones, 1981; Alma et al., 2005), with late sowing generally associated with a higher content of mycotoxins at harvest (Alma et al., 2005; Blandino et al., 2009; Mazzoni et al., 2011). Nonethless, irrigation has a strong impact as well, particularly upon AFs occurrence (Palumbo et al., 2020). Both the severity of European corn borer (ECB) (Jones, 1981) and the use of insecticide treatments may also significantly affect contamination, especially from FBs (Alma et al., 2005; Saladini et al., 2008; Mazzoni et al., 2011). Harvest time, or rather the kernel moisture at harvest, can also be crucial, notably for AFs contamination of maize (Munkvold, 2003; Battilani et al., 2008a); in fact, AFs production increases significantly from maize physiological maturity, when kernel moisture is lower than 28–30% (Payne et al., 1988; Giorni et al., 2016). Therefore, delays in maize harvest after that stage means giving the fungus time to efficiently increase the contamination. Then, keep kernel moisture below 14% is mandatory in the postharvest stages, from drying to the whole storage period (Danso et al., 2018).

The above research findings have contributed to developing guidelines for mitigating mycotoxin contamination, but a quantitative evaluation of cropping system’s impact on mycotoxins remains an unresolved issue. The little work done so far to predict the effect of cropping system on mycotoxin contamination (Battilani et al., 2008b; Camardo Leggieri et al., 2015) is neither complete nor satisfactory; however, it does clarify that cropping factors cannot be considered in isolation and that applying conventional statistical methods is not suitable for the task (Battilani, 2016; Palumbo et al., 2020). Hence, alternative approaches should be explored and possibly used.

Machine learning’s emergence, alongside big data technologies and high-performance computing, introduces new opportunities for data-intensive science in precion farming and sustainable agriculture (Liakos et al., 2018). ML is the scientific field in which machines are trained to learn without being strictly programmed (Samuel, 2000), which has three main categories: (1) supervised learning (SL), (2) unsupervised learning (UL), and (3) reinforcement learning (RL). The SL algorithms use a training data set of labeled data to infer a function that is used to map new data. The UL algorithms directly look at the data and learn patterns from them, without human supervision. Last is RL, the ML branch in which entities called “software agents” take action, in a specific context, to optimize a given function. These ML approaches are increasingly applied in different subject areas to solve complex problems, often those with many factors involved, to which agriculture is no exception. In fact, ML is used in a variety of contexts and all the three main categories are now applicable (Liakos et al., 2018; Elavarasan and Vincent, 2020). Recently, Liakos et al. (2018) reviewed the ML approach in agriculture, highlighting that ML models had been applied in the multi-disciplinary agri-technologies domain for crop management (61%), yield prediction (20%), and disease detection (22%), but never accounting specifically for mycotoxins’ co-occurrence. In crop yield prediction, which depends on many different factors operating simultaneously, deep neural networks (DNN), a type of artificial neural network (ANN) for SL models, are the most used (Khaki and Wang, 2019; Khaki et al., 2019; Nevavuori et al., 2019; Niedbała, 2019). DNNs are also very useful for plant disease identification, which is done via convolutional neural networks (CNN), which is a specific DNN-architecture used for image recognition (Boulent et al., 2019). Mycotoxins are mainly detected via high-performance liquid chromatography (HPLC) and mass spectrometry; however, DNNs coupled with rapid analytical tools, such as the electronic nose or infrared attenuated total reflection spectroscopy, have been recently applied and found to improve the assessment reliability (Evans et al., 2000; Jia et al., 2019; Öner et al., 2019; Camardo Leggieri et al., 2020b).

Torelli et al. (2012), in the first example of ML applied to mycotoxins, performed a 2-year study (2007–2008) that included seven cropping system variables—FAO class, sowing and harvest dates, crop duration, kernel moisture, ECB treatment, and irrigation—as input for an ANN to classify maize samples based on their contamination with FBs. A fair correlation between the predicted and observed contaminated samples was reported (*R*^2^ = 0.67 and *R*^2^ = 0.57, respectively, for the training and validation data sets), so the approach seems promising.

Therefore, this study aimed to develop ML models by combining the AFLA-maize and FER-maize predictions, together with cropping system information, as input to improve the mycotoxin risk predictions for AFB_1_ and FBs. For this, A 13-year data set was considered and two ML models, one for each type of mycotoxin, were trained and validated (5-fold cross-validation) and their respective performance discussed.

## Materials and Methods

### Data Collection

The data used in this study came from several surveys managed in the Emilia Romagna region (northern Italy) during 2004–2018, partially published (Battilani et al., 2013; Camardo Leggieri et al., 2015, 2020b). The protocol for data collection was the same both for the published and unpublished data.

Briefly, meteorological data were downloaded from the Emilia Romagna meteorological service, available on request for research applications. They were based on a grid of squares, each 5 km wide, that encompassed the Emilia Romagna territory; all sources (both meteorological stations and radar) are interpolated for each square, to deliver reliable data (Bottarelli and Zinoni, 2002). Hourly data on air temperature (T, °C), relative humidity (RH, %), and rain (R, mm), during the period of January through September, were downloaded. These squares were filtered, to locare those corresponding to the maize field site sampled.

Maize fields sampling was performed at harvest, managed between mid August and September, during the combine machine discharge, according to European Commission Regulation (EU) 401/2006 (European Commission, 2006a). Relevant cropping system data were collected in each georeferenced field, based on a questionnaire filled by farmers, supported by extension services. Empirical information of different site variables were collected: the type of soil (percentages of sand, clay, and silt), maize hybrid FAO class, preceding crop, type of tillage, sowing date, plants per m^2^, silk emission date, harvest date, damage caused by hail or wind and ECB, fertilization type and dose, the number of irrigation intervention with relative volumes of water used, pest and disease control practices, and kernel moisture at harvest. Mycotoxin analysis was performed for all the sampled fields according to Bertuzzi et al. (2012) for the AFs [limit od detection (LOD): 0.05 μg/kg and limit of quantification (LOQ): 0.15 μg/kg], and by following Pietri and Bertuzzi (2012) for the FBs (LOD: 10 μg/kg and LOQ: 30 μg/kg).

### Data Processing

AFB_1_ and FBs content allowed in maize grain, according to legal limits, were used as a threshold to separate the field samples in two classes: (1) *contaminated*, consisting of those samples equal or exceeding the respective legal limit; (0) *non-contaminated* comprising all samples below the legal limit. Thresholds were therefore set to 5 μg/Kg for AFB_1_ and 4,000 μg/Kg for FBs (FB_1_ + FB_2_), the legal limits currently set by the European Commission for unprocessed maize destined for human consumption (European Commission, 2006b, 2007).

Meteorological data were used as input for the two predictive models, AFLA-maize and FER-maize, and cumulative risk indexes were obtained as the output, AFI for AFB_1_ and FK for FBs, for each station and year. DNN models were implemented within the frame of Scikit-Learn (v0.21.3) in the Python module library (Pedregosa et al., 2011).

### Input Features

After the exclusion of those variables with many missing data points, eight different variables were considered as input for the ML approach; sowing date and harvest date were grouped on a per week basis. Of these eight, five variables were categorical (maize hybrid FAO class, preceding crop, sowing week, harvest week, ECB damage; Table 1) and three were continuous variables (growing days, days from crop sowing to harvest, calculated variable, kernel moisture at harvest, and mycotoxin cumulative indices AFI and FK, the output of predictive models).

**TABLE 1:** Summary of categorical data used for the two pathosystems analyzed: *A. flavus*-maize and *F. verticilloides*-maize.

**Variable**	**N. of categories**	**Categorical value**	**Integer encoding**
Maize hybrid FAO class	4	200–300	1
		400	2
		500	3
		600–700	4
Preceding crop	3	arable crops	1
		small grain	2
		maize	3
Sowing week*	4	10–12	1
		13–14	2
		15–16	3
		17+	4
Harvest week*	4	32–35	1
		36–37	2
		38–39	3
		40+	4
Severity of ECB attack	3	No/Minor-damage	1
		Medium damage	2
		Severe damage	3

Variables (hybrid FAO classes, preceding crop, and sowing and harvest weeks of the year (Julian date) and European corn borer [ECB] damage) and numbers of utilized categories are reported. *n^*th*^ week of the year.

For each continuous variable, its average (μ) and standard deviation (σ) were computed for the data standardization, using Eq. (1).

(1) $$X=\frac{X_{0}-\mu }{\sigma }$$

Even if this procedure is entirely facultative in neural network training, it is nonetheless useful for reducing the variance, speeding up the computational process, and improving the model’s accuracy (Jin et al., 2015).

To deal with categorical data, the integer encoding procedure was applied. This assigns to a specific category an integer value that ranges from 1 to N, where N is the last category.

### Deep Neural Network (DNN) Development

A typical ANN consists of a network of connected computational units called neurons; these units are organized in layers, with input data passing through the network, and an activation function used to produce an output. DNNs are a particular class of ANNs in which, between the input and the output layer, there is an arbitrary number of hidden layers. The fully connected architecture has been adopted, meaning that each neuron in each layer is connected to each neuron in the next adjacent layer.

The development of a DNN model is a two-step process: (i) training and (ii) validation. The final aim of the training is to minimize a given error function by using an optimization algorithm. The training phase ends when the error converges to a pre-determined value, or when it does not decrease for a specific number of cycles, both decided *a priori* by the user (Camardo Leggieri et al., 2020b). A “batch training” mode was applied in this work. Briefly, during the training phase, training data (i.e., the mycotoxin contamination data in this study), were split into subgroups called *batches*. Neural network weights are updated when every sample inside a batch passes through the network. The iteration ends when all batches have passed through the network. After each iteration, a penalization term, called the weight decay (L2 regularization term), is introduced into the model to avoid overfitting the model to the data.

The final output is the result of a linear or non-linear activation function. In our study, a non-linear activation function between the input layer and the hidden layers, called Rectified Linear Unit (ReLU, Eq. 2), was applied:

(2) $$f\,\left(x\right)=\left\{ \begin{array}{c} 0,x\leq 0\\ x,x>0 \end{array}\right.$$

where, *x* represents the weighted sum in a given input to a neuron (Dahl et al., 2013; Zeiler et al., 2013; LeCun et al., 2015).

The activation function used between the last hidden layer and the output layer (classification function) took the logistic form (Eq. 3):

(3) $$f\,\left(x\right)=\frac{1}{1-e^{-\vartheta \,\sum_{i=1}^{N}x_{i}\,w_{i\,j}}}$$

where, *j* is relative to the *j*^*th*^ output neuron, and *i* represents the *i*^*th*^ input neuron. The numerical result is between 0 and 1, for which 0.5 served as a threshold to discriminate between the positive and negative classes.

Different DL models were tested following a grid search procedure, done as described in Camardo Leggieri et al. (2020b). Briefly, each hyperparameter was tested with a combination of every other hyperparameter. The hyperparameters tested were weight decay, number of hidden layers, number of neurons per hidden layer, and the optimization algorithm. In this work, two algorithms where tested; the first approximates the Broyden–Fletcher–Goldfarb–Shanno algorithm and is called LBFGS (Byrd et al., 1995; Kingma and Ba, 2014), while the second is an optimization of the classical stochastic gradient descent, called Adam (Kingma and Ba, 2014). Matthew’s correlation coefficient (MCC) and accuracy were used as metrics to select the best combination of hyperparameters, for both NN models relevant to the pathosystem *A. flavus*-maize (DNN-*A. flavus*-maize) and *F. verticillioides*-maize (DNN-*F. verticilloides*-maize).

### DNN Validation

As in Camardo Leggieri et al. (2020b), both AFB_1_ and FBs original datasets were splitted into two “sub-dataset.” These four “sub-datasets” (two for AFB_1_ and two for FBs) were generated by random sampling, but keeping the proportion of contaminated vs. non-contaminated samples constant. The first “sub-data set” accounted for 75% of the original data set and was used to perform a 5-fold cross-validation (CV). The other, called the “blind set” in this work, accounted for the rest of the original data set (25% of the original data set), and was used for a further validation of the models. The goodness-of-fit of each DNN-*A. flavus*-maize and DNN-*F. verticilloides*-maize model was assessed by computing several statistical scores, which were also applied to the blind set:

• True positive and true negative rates (TPR, TNR; Kohavi and Provost, 1998);

(4) $$T\,N\,R=\frac{T\,N}{T\,N+F\,P}$$

where, TP and TN denote the number of true positives and true negatives, respectively, and likewise FP and FN denote the number of false positives and false negatives.

• Positive predictive value (PPV): the PPV (Eq. 5) index represents the proportion of positives samples identified as true positives. It ranges from 0 to 1 (Kohavi and Provost, 1998)

(5) $$P\,P\,V=\frac{T\,P}{T\,P+F\,P}$$

• *Receiver operator characteristic* (ROC) curve and *area under the curve* (AUC): the ROC curve and its AUC measure the quality of a binary classifier: the higher the area under the curve, the better the model performs. The AUC value ranges from 0 to 1(Bradley, 1997).
• The MCC (Eq. 6) is used to assess the quality of binary classification (Matthews, 1975). This index takes into consideration TPR, TPN, and both false discoveries (false positives and negatives). The MCC ranges between –1 (complete disagreement between predicted and observed values) and +1 (perfect agreement). The MCC is considered a balanced measure, and it can be used even if the two classes differ in size (Boughorbel et al., 2017).

(6) $$M\,C\,C=\frac{T\,P\times T\,N-F\,P\times F\,N}{\sqrt[{2}]{\left(T\,P+F\,P\right)\,\left(T\,P+F\,N\right)\,\left(T\,N+F\,P\right)\,\left(T\,N+F\,N\right)}}$$

All the scores were computed using a home-built Python (v3.6.9) script that implemented the equations reported above. The ROC curves and AUCs, the grid search procedure, the MCCs, and the DNN architecture were implemented in the framework of scikit-learn (v0.23.2; Pedregosa et al., 2011).

The output indexes of the two mechanistic models (AFI and FK) were used to classify the blind data set, as described in Battilani et al. (2003) for the FBs and in Battilani et al. (2013) for AFB_1_. Finally, the results were compared using the classification obtained by the two DNNs.

## Results

A total of 378 and 225 samples were included in the *A. flavus*-maize and *F. verticilloides*-maize data sets, respectively (Table 2). No data were retrieved for FBs before 2009 or during 2012 and 2013. Concerning the AFB_1_, data for it were not retrieved for the years 2012 and 2013. The sample sizes per year were slightly different for the two data sets because of this missing data.

**TABLE 2:** Descriptive statistics of aflatoxin B1 (AFB_1_) and fumonisins (FBs, intended as the sum of FB_1_ + FB_2_) levels of contamination (μg/kg) in maize grain samples collected in Emilia Romagna, Italy, over the years 2005–2018 (with some exceptions both for AFB_1_ and for FBs).

**Data set**	**Year**	N.*	**%positives^§^**	**Mean**	**StDev**	**Minimum**	**Maximum**
**AFB_1_**							
	2005	70	41.4	13.8	29.65	<0.05	154.9
	2006	25	24.0	18.8	52.78	<0.05	258.3
	2007	29	27.6	8.34	18.50	<0.05	68.43
	2008	40	40.0	9.11	16.99	<0.05	93.79
	2009	31	29.0	23.3	88.51	<0.15	494.3
	2010	35	28.0	14.4	36.60	<0.05	173.3
	2011	31	12.9	14.9	61.03	<0.05	334.8
	2014	26	23.1	10.2	23.17	<0.15	93.77
	2015	15	20.0	11.2	32.34	<0.05	129.3
	2016	20	45.0	30.4	62.33	0.42	208.3
	2017	28	50.0	22.3	29.15	<0.05	116.2
	2018	28	25.0	5.76	13.09	<0.05	65.00
	Total	378	35.54	14.66	42.59	<0.05	494.3
**FB_1_ + FB_2_**							
	2009	31	16.1	2,721.93	2,423.335	139.3	8,829.7
	2010	36	40.0	3,975.79	3,221.642	142.7	12,637.0
	2011	30	6.67	2,344.87	3,689.235	74.0	21,007.0
	2014	45	84.4	17,586.88	18,300.650	1718.4	106,053.5
	2016	21	19.0	3,035.33	3,251.853	204.3	14,020.8
	2017	29	13.8	2,643.91	3,107.761	<10.0	14,767.4
	2018	33	21.2	3,846.22	6,298.140	51.8	29,632.7
	Total	225	38.22	6,029.34	10,566.241	<10.0	106,053.5

The percentage of positive samples above the legal limits (5 and 4,000 μg/kg for AFB_1_ and FBs, respectively; European Commission, 2006a, 2007) is reported. *N. = number of samples collected in the year; ^§^ % of positive = % of fields with contamination above the legal limits.

Fields with AFB_1_ contamination above 5.0 μg/kg were found, whose incidence and mean values differed across the considered years. The highest amount of AFB_1_ was found in 2009, at 494.3 μg/kg. In all years AFB_1_ was greater than LOQ, but lower than LOD, except in 2009, 2014, and 2016. Regarding the incidence of fields found positive for AFB_1_, this was highest at 50% in 2017 and the lowest (12.9%) in 2011 (Table 2). In the whole maize data set, for AFB_1_, the mean incidence of positive samples was 32.9%.

Fields with FBs’ contamination above 4,000 μg/kg were found in all the years considered, but this incidence differed across all years. The only year when the FBs was below the LOD was in 2017. The highest amount of FBs (106,053.5 μg/Kg) and the highest incidence of positive samples (89.8%) were both detected in 2014. The lowest incidence of FBs was 6.7%, scored in 2011 (Table 2). Considering the FER-maize data set as a whole, the overall mean incidence of positive samples, above the legal limit, was 32.2%.

Means and standard deviations computed for AFI and FK, the kernel moisture, and the growing days, are reported in Table 3.

**TABLE 3:** Basic statistics of the continuous data included as the input into the model for the two pathosystems, *A. flavus*-maize and *F. verticillioides*-maize.

**Model**	**Variable**	**Mean**	**StDev**	**Maximum**	**Minimum**
*A. flavus*-maize	AFI index	2,906.17	2,226.792	8,944.5	11.6
	Kernel moisture (%)	20.66	3.541	31.5	11.9
	Growing days	158.0	16.68	234	66
*F. verticilloides*-maize	FK index	246,407.4	626,994.91	537,8545.3	2,102.3
	Kernel moisture (%)	19.99	3.712	31.5	11.8
	Growing days	156.6	16.30	207	115

Categorical data were grouped into three or four categories (Table 1). The FAO class was represented by four categories: 200–300, 400, 500, and 600–700. The preceding crop was represented by three categories: arable crops, small grain, and maize. The sowing and harvest weeks both accounted for four categories. Considering the sowing week, category #1 was assigned to weeks 10–12 of the year, #2 to weeks 13–14, #3 to weeks 15–16 and #4 to week ≥ 17. For the harvest week, those weeks of the year from 32 to 35 were designated category #1, and likewise 36–37 to # 2, 38–39 to #3, with all weeks > 40 assigned to #4. The damage caused by ECB was divided into three categories: no damage and small damage were grouped into category #1, medium damage was assigned to category #2, and severe damage was assigned to category #3 (Camardo Leggieri et al., 2015).

Standardized continuous data were joined to categorical data, to form the neural network’s input vector; thus, the final input array was formed by five encoded and three continuous variables (Tables 1 and 3), respectively.

The generation of the “sub-datasets” included a total 283 (75%) and 95 (25%) samples in the CV and blind set respectively for AFB_1_, and 169 and 56 samples in the CV and blind set respectively for FBs.

### Deep Neural Network (DNN) Training

The two DNNs were trained to be able to predict the content of AFB_1_ and FBs, respectively. The NN-*A. flavus*-maize model was implemented with one hidden layer consisting of 80 neurons, a ReLU activation function, and an L2 regularization term of 0.0001. The parameters of that model were updated using the Adam algorithm (Kingma and Ba, 2014). By contrast, the NN-*F. verticilloides*-maize model’s sole hidden layer had 50 neurons, a ReLU activation function, and an L2 regularization term of 0.1. Its parameters were updated using the LBFGS algorithm (Byrd et al., 1995; Table 4). The two developed NN models were validated using both the 5-fold cross-validation and a blind data set.

**TABLE 4:** Hyperparameter values used to implement the neural networks *A. flavus*-maize (NN-*A. flavus*-maize) and *F. verticilloides*-maize (NN-*F. verticilloides*-maize) models.

**Hyperparameter**	***NN-A. flavus*-maize**	***NN-F. verticilloides*-maize**
Number of input neurons	7	7
Number of hidden layers	1	1
Number of neurons per hidden layer	80	50
Activation function input—hidden	ReLU (Eq. 1)	ReLU (Eq. 1)
Activation function hidden—output	Logistic (Eq. 2)	Logistic (Eq. 2)
L2 regularization term	0.0001	1.024
Parameters update algorithm	Adam	LBFGS

**The number of input neurons is defined as the sum of the categorical and continuous data. The hyperparameter values were selected by following a grid searching procedure.**

### DNN Validation

**FIGURE 1:**
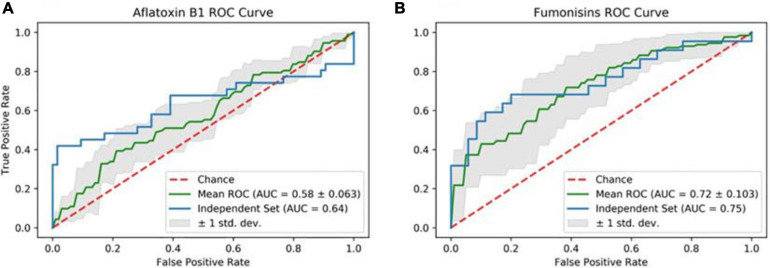
Receiver operating characteristics (ROC) curves for the independent data set for the **(A)** aflatoxin B1 and **(B)** FBs models. The solid blue lines represent the ROCs for the two models. The goodness-of-fit of the models is conveyed as the area under the curve (AUC): the higher it is, the better the model performed. The dotted red line represents the random prediction.

Cross-validation ROC curves and their relative AUCs were computed for the two NN models, to assess the quality of the two classifiers (Figures 1A,B). The 5-fold-cross-validation for NN-*A. flavus*-maize achieved an accuracy of 66.56 ± 3.381 (mean ± SE), and even higher at 78.94 for the blind data set. An MCC of 0.10 ± 0.157 and 0.49 were achieved by the cross-validation and blind data set, respectively. The AUC and the TPR averaged 0.58 ± 0.063 and 0.08 ± 0.073 during the model’s cross-validation. The model scored an AUC of 0.64 and a TPR of 0.42 when tested against the blind data set (Table 5).

**TABLE 5:** Classification results summary for the prediction of aflatoxin B_1_ (AFB_1_) and fumonisins (intended as the sum of FB_1_ + FB_2_, FBs) in the maize samples.

	**CV**	**Blind data set (DNN-models)**	**Blind data set (mechanistic models)**
**AFB_1_**			
ACC	66.56 ± 3.381	78.94	52.63
TPR	0.08 ± 0.073	0.42	0.32
TNR	0.94 ± 0.053	0.96	0.63
PPV	0.59 ± 0.424	0.90	0.29
MCC	0.10 ± 0.157	0.49	–0.051
**FBs**			
ACC	69.63 ± 10.892	79.31	52.63
TPR	0.53 ± 0.118	0.65	0.81
TNR	0.80 ± 0.122	0.88	0.34
PPV	0.63 ± 0.175	0.78	0.44
AUC	0.72 ± 0.103	0.75	n.c.
MCC	0.35 ± 0.229	0.56	0.17

**The deep neural networks (DNNs) considered were evaluated using a 5-fold cross-validation and a blind data set. Cross-validation (CV) results were reported as an average value and related standard deviation (Avg ± Stdev). ACC, accuracy; MCC, Matthew’s correlation coefficient; AUC, area under the curve; TPR, true positive rate; TNR, true negative rate; PPV, positive predictive value; n.c., not computed.**

The NN-*F. verticilloides*-maize model’s 5-fold cross-validation attained an accuracy of 69.63 ± 10.892; the accuracy was 79.31% using the independent data set. A TPR of 0.53 ± 0.118 and 0.65 were, respectively, achieved by cross-validation and blind data set, respectively. Moreover, the model had an AUC of 0.72 ± 0.103 and an MCC of 0.35 ± 0.229 during the cross-validation phase, with corresponding values of 0.75 and 0.56 when tested against the unseen data set (Table 4).

Finally, to check whether the new approach represented a major step forward in the prediction of mycotoxin contamination in maize, our two DNN-models were compared with two counterpart mechanistic models, both run with the whole available data set. The resulting confusion matrix (Table 6) shows the performances of the NN-*A. flavus*-maize and NN-*F. verticilloides*-maize models and those of the two mechanistic models vis-à-vis the blind data set. The NN-*A. flavus*-maize model correctly estimated about 78% of samples (14% true positives, 64% true negatives). The wrong classification accounted for 19% of them being underestimated and 3% overestimated. The NN-FER-maize model correctly classified approximately 75% of the data set (25% true positives, 50% true negatives), with underestimations and overestimations amounting to 15 and 11%, respectively. In stark contrast, the AFLA-maize model correctly predicted just ∼53% of samples (11% true positives, 42% true negatives). Further, a wrong classification accounted for more underestimations (22%) and overestimations (25%). Similarly, the FER-maize model correctly classified only ∼52% of samples (31% true positives, 20% true negatives), but its wrong classifications included fewer (7%) underestimated cases being more prone to overestimations (41%).

**TABLE 6:** Confusion matrix computed from the blind data set results for the predicted and observed values of aflatoxin B1 (AFB_1_) and fumonisins (intended as the sum of FB_1_ + FB_2_, FBs). The predicted vs. observed results are reported as percentages.

**DNNs**		**Predicted**	
	**Observed**	**Negative**	**Positive**
AFB_1_	Negative	65	2
	Positive	19	14
FBs	Negative	53	7
	Positive	14	26
**Mechanistic models**			
AFLA-maize	Negative	42	25
	Positive	22	11
FER-maize	Negative	21	41
	Positive	7	31

## Discussion

Maize is exposed to mycotoxins, which threaten human and animal health, and represent the major non-tariff trade barrier for agricultural products, negatively affecting the income of small-holder farmers and disrupting regional and international trade (Palumbo et al., 2020; Logrieco et al., 2021). Timely identification of contaminated lots is not a trivial challenge since mycotoxin contamination relies on several factors, including meteorology and how farmers manage the crop during the season and in the postharvest stages of storage and distribution (Munkvold, 2014; Logrieco et al., 2021). Different methodologies for the rapid detection of mycotoxin contamination are currently available (Öner et al., 2019; Camardo Leggieri et al., 2020b), but since they are applied at harvest or postharvest stages, they offer no support for taking preventive action and for optimizing lot use and management. On the contrary, farmers can benefit from model predictions of the risk of mycotoxin occurrence above the legal limit, when delivered before or during the cropping season, in the form of risk maps or risk indexes. Therefore, predictive modeling has garnered mounting interest over the last two decades (Battilani, 2016). Predictions refer to maize at harvest and it is assumed that the postharvest management guarantee a rapid grain drying to humidity ≤14%, kept stable during storage, to avoid fungal activity and further mycotoxin production.

Meteorological factors jointly determine whether fungi can grow and produce toxins, while the site’s cropping system modulates the amount of contamination that ensues (Battilani and Camardo Leggieri, 2015). The former are the driving variables for predictive modeling, whereas the latter are rarely included, especially in mechanistic models. This omission is starting to gravely limit the reliability of predictions; in fact, during the last two decades, the typical cropping system has changed significantly due to the knowledge transfer from scientists to farmers; farmers are now following the guidelines to optimize crop management and mitigate mycotoxin contamination, with good results so far in term of a reduced mycotoxin occurrence. Both the meteorological data and the cropping system data have been used before as model inputs, to predict the content of mycotoxin in maize at the time of its harvest (Battilani et al., 2003, 2008a; Bertuzzi et al., 2014; Camardo Leggieri et al., 2015), yet they were used independently and only supported by basic statistical approaches. The aim of this study was to evaluate how combining cropping system information with mechanistic predictive models could support the sought-after improvement in prediction performance.

Here an ML approach was developed using the AFLA-maize and FER-maize outputs (mycotoxin risk indexes) combined with cropping system information—this being known to significantly influence mycotoxin contamination in maize according to other studies (Palumbo et al., 2020; Logrieco et al., 2021)—as input variables. Other crop-related variables should have been included, like fertilization, irrigation, and pest control (Mazzoni et al., 2011; Munkvold, 2014); however, we excluded them because this data was largely unavailable to us. Moreover, the geolocation of maize fields was excluded as an input variable in our modeling; actually, even when the field location is known to be relevant (Torelli et al., 2012; Camardo Leggieri et al., 2015), the idea of this work was to obtain models applicable at a global level, without geographical constraints. When combining weather data and cropping system no information is lost, even when the maize fields’ geolocation is excluded.

The predictions of NN-*A. flavus*-maize and NN-*F. verticilloides*-maize, the two neural network models developed in this study, were capable of an accuracy approaching ∼78% for AFB_1_ and ∼79% for FBs, with a good correlation between predicted and observed data. This result is supported by the MCC results, which reached values of 0.49 and 0.56 when computed for the NN-*A. flavus*-maize and NN-*F. verticilloides*-maize, and by their corresponding AUC of 0.64 and 0.75 for NN-*A. flavus*-maize and NN-*F. verticilloides*-maize. Both AFLA-maize and FER-maize, the mechanistic models which served as the starting point of our investigation, achieved accuracies one-third lower, of about 50%, and their respective MCC was very close to 0; this indicates that their predictions were comparable to random one, when based on the same data set (i.e., the blind data set) used for NN model evaluation. It is, therefore, evident the proposed ML approach significantly improved the prediction of mycotoxins’ content across the studied maize fields, making the successful use of this tool to detect maize grain not compliant with the current legal limit in Europe now more realistic and feasible to implement. Nevertheless, the NN-*F. verticillodes-*maize model performed better than NN-*A. flavus-*maize; apparently, it is easier to predict levels of FBs than AFB_1_ as contaminants. The reason for this is not entirely clear, but the very low limit fixed by the legislation for AFB_1_ could surely play a role. Further, irrigation is known to be very relevant for AFB_1_ contamination, and grain humidity at harvest too, but they were excluded as input variables because of many missing data and this has surely a considerable impact on the predictive capacity of the model.

The NN-models were developed based on a large data set, one that included data collected from over 12 different years, with 378 samples in the AFB_1_ data set and 225 samples in the FBs data set. The large data sets used, and the range of years considered, including the period of significant changes in the cropping system, strongly support the robustness of NN-*A. flavus*-maize and NN-*F. verticilloides*-maize models and their promising utility as a tool to support farmers in their decision-making. Future applications in other pathosystems is also foreseeable, as previously done for AFLA-maize (Kaminiaris et al., 2020).

## Conclusion

To conclude, despite omitting some relevant cropping system variables, a substantial improvement at correctly predicting maize fields contaminated with mycotoxins above their legal limits was gained. Further improvements should be obtained by optimizing the data collection. Solving the missing data problem might be an easy task in the future, once scientists succeed in convincing farmers of the crucial role they can play in such data collection and also of the added value of predictive models in mycotoxin management. This will be a matter of building with the maize chain stakeholders a knowledge exchange approach and make them more involved compared to what actually happened in the past. Another aspect of improvement could be gleaned through our modeling approach, that of emerging issues related to mycotoxins. The main example of this concerns the recent report of co-occurring *A. flavus* and *F. verticilloides* in maize ears due to climate change effects, resulting in their complex interaction, with their dominance alternating during the growing season (Camardo Leggieri et al., 2019, 2020a; Giorni et al., 2019). Looking ahead, we anticipate that elucidating the impact of these interactions between co-existing fungi upon mycotoxin production in maize will become crucial. Data collection to develop a joint model for the prediction of AFB_1_ and FBs, including the impact of fungi co-occurrence, is ongoing and in the next future it is expected to contribute to a step up in mycotoxin prediction, possibly joining also NN-*A. flavus*-maize and NN-*F. verticilloides*-maize in a NN-mycotox-maize predictive model.

The current work represents a notable step forward in modeling and predicting mycotoxins in crops. We retrieved evidence that ML can effectively combine cropping system data and meteorological data, thereby improving the accuracy and robustness of predictions. Big data is a relatively new concept for agriculture and plant disease research and management, but massive volumes of data with several components that interact within the pathosystem can also be captured in this context, and their elaboration can enhance the decision-making process (Wolfert et al., 2017). Applying machine learning to farm management systems is quickly evolving into a real artificial intelligence (AI) system, providing richer recommendations and insight for subsequent decisions and timely actions (Liakos et al., 2018). Further research that aims to integrate automated data recording, mycotoxin analysis, ML implementation, and decision support systems will provide practical tools in line with so-called “knowledge-based agriculture.” This should move us closer toward sustainable agriculture and smart farming that also improves food safety and quality.

## Data Availability Statement

The raw data supporting the conclusions of this article will be made available by the authors, without undue reservation, to any qualified researcher.

## Author Contributions

PB, MC, and MM contributed to the conception and design of the study and wrote sections of the manuscript. MC organized the database. MM performed the statistical analysis. MC and MM wrote the first draft of the manuscript. All authors contributed to manuscript revision, read it, and approved the final submitted version.

## Conflict of Interest

The authors declare that the research was conducted in the absence of any commercial or financial relationships that could be construed as a potential conflict of interest.
